# Identification of FTO as a key m6A demethylase linking immune dysregulation to sepsis pathogenesis

**DOI:** 10.3389/fimmu.2026.1756059

**Published:** 2026-02-18

**Authors:** Yi Jiao, Rui Lian, Weijian Zhang, Nan Gao, Qishun Geng, Tiantian Deng, Zhaoran Wang, Tingting Deng, Cheng Xiao, Guoqiang Zhang

**Affiliations:** 1 Department of Emergency, China-Japan Friendship Hospital, Beijing, China; 2 Institute of Clinical Medical Sciences, China-Japan Friendship Hospital, Beijing, China; 3 China-Japan Friendship Hospital, Chinese Academy of Medical Sciences & Peking Union Medical College, Beijing, China; 4 Department of Pharmacy, The First Affiliated Hospital of Zhengzhou University, Zhengzhou, Henan, China; 5 Beijing University of Chinese Medicine, School of Clinical Medicine, China-Japan Friendship Hospital, Beijing, China

**Keywords:** FTO, M1 macrophages, N6-Methyladenosine, neutrophils, sepsis

## Abstract

**Background:**

Sepsis is a life-threatening disorder characterized by multiple organ dysfunction caused by dysregulated host responses to infection. The present study aimed to identify potential diagnostic biomarkers for sepsis and elucidate their molecular mechanisms through comprehensive bioinformatics and experimental analyses.

**Methods:**

Five publicly available transcriptomic datasets (GSE13904, GSE26440, GSE28750, GSE95233, and GSE57065) containing sepsis and healthy control samples were utilized in the study. After quality control and normalization, the samples were divided into training and validation cohorts. Fourteen machine learning algorithms were applied to the training cohort to identify robust diagnostic biomarkers, and their predictive performance was subsequently verified in the validation cohorts. Single-cell RNA sequencing (scRNA-seq) data were further analyzed to determine the cellular distribution of the identified regulators among immune cell subsets.

**Results:**

In total, the least absolute shrinkage and selection operator (LASSO) model exhibited the best performance in the validation set, demonstrating high reliability. Through consensus feature selection across multiple models, the m^6^A methylation regulator fat mass and obesity-associated protein (FTO) was identified as a key biomarker. scRNA-seq analysis revealed that FTO was primarily expressed in neutrophils and macrophages. Its expression levels were markedly altered in peripheral blood mononuclear cells (PBMCs) and neutrophils from sepsis patients compared with healthy controls, which was consistent with the findings in in vitro macrophage and neutrophil models. Functional experiments demonstrated that FTO promotes macrophage polarization toward the pro-inflammatory M1 phenotype and enhances neutrophil inflammatory and chemotactic responses, highlighting its critical role in orchestrating inflammatory regulation during sepsis.

**Conclusion:**

FTO, identified through consensus machine learning approaches, could serve as a potential diagnostic biomarker and m^6^A methylation regulator for sepsis. The discovery of FTO and its downstream targets provides new insights into sepsis pathogenesis and may offer a foundation for developing novel therapeutic strategies.

## 1 Introduction

Sepsis is a complex clinical syndrome characterized by a dysregulated host response to infection, leading to life-threatening multiorgan dysfunction. This maladaptive immune response not only triggers sustained hyperinflammation but also may result in multiorgan failure, septic shock, and even death. The spectrum of sepsis-associated organ dysfunction includes endothelial dysfunction, cardiac impairment, acute lung injury, acute kidney injury, and liver failure (1, 2). According to data from 2017, approximately 48.9 million sepsis cases occur globally each year, with 11 million sepsis-related deaths, accounting for 19.7% of all annual deaths worldwide (3), making sepsis one of the most severe medical challenges globally. The pathogenesis of sepsis is highly complex and involves both early excessive inflammation and concurrent or subsequent immunosuppression, which are frequently accompanied by endothelial dysfunction, coagulation abnormalities, and metabolic disturbances (4, 5). Although recent advances have improved our understanding of these pathological processes, many critical mechanisms remain incompletely understood. Therefore, further investigations into the pathogenesis of sepsis are essential for enhancing our understanding of this disease and facilitating the development of effective therapeutic strategies.

In the pathophysiological process of sepsis, dysregulated immune responses represent a central mechanism (6). Patients frequently exhibit a combination of hyperinflammation and immunosuppression (7), leading to considerable immune dysfunction. These alterations include enhanced lymphocyte apoptosis, impaired antigen-presenting cell (APC) function, excessive release of anti-inflammatory cytokines such as interleukin-10 (IL-10), and upregulation of immune checkpoint molecules, including programmed cell death protein 1 (PD-1) and programmed death-ligand 1 (PD-L1) (8–10). Moreover, the innate immune system of septic patients often exhibits defective neutrophil chemotaxis and bactericidal activity and impaired macrophage phagocytosis and antigen presentation, whereas the adaptive immune system is characterized by a notable reduction in lymphocyte counts and functional exhaustion (6, 11). Although the pathogenesis of sepsis has not been fully elucidated, immune cells play a pivotal role in shaping its initiation and progression (12). Therefore, a systematic investigation of immune cell infiltration and functional alterations in sepsis is essential for advancing our understanding of its pathophysiology and for guiding the development of effective therapeutic strategies.

With the continuous advancement of epigenetic research, the focus has gradually expanded from DNA and histone modifications to RNA modifications. Among them, N^6^-methyladenosine (m^6^A) is the most prevalent internal modification of eukaryotic mRNA, accounting for more than 80% of all RNA methylation events. Notably, m^6^A is widely distributed across the eukaryotic transcriptome and has emerged as a major focus of biomedical research (13). m^6^A modification is involved in multiple critical stages of the RNA life cycle, including pre-mRNA splicing, nuclear export, translational regulation, and the control of mRNA stability and degradation, thereby greatly increasing the complexity of gene expression regulation (14). In addition to mRNA, m^6^A modification also targets a wide range of non-coding RNAs, including microRNAs, circular RNAs, and long non-coding RNAs, affecting their processing, stability, and cellular localization, which further broadens the regulatory potential of m^6^A in complex biological processes and disease contexts such as cancer, immunity, and inflammation (15). The dynamic regulation of m^6^A is mediated by three classes of factors: writers [methyltransferase-like 3 (METTL3), METTL14, Wilms tumor 1–associated protein (WTAP), zinc finger CCHC-type containing 4 (ZCCHC4), KIAA1429 (also known as VIRMA), RNA binding motif protein 15 (RBM15), RNA binding motif protein 15B (RBM15B), and tRNA methyltransferase 112 (TRMT112)]; erasers [fat mass and obesity-associated protein (FTO), alkB homolog 5 (ALKBH5), and ALKBH3]; and readers [YTH domain family proteins (YTHDF family), YTH domain containing proteins (YTHDC family), insulin-like growth factor 2 mRNA-binding proteins (IGF2BP family), heterogeneous nuclear ribonucleoproteins (HNRNP family), leucine-rich pentatricopeptide repeat-containing protein (LRPPRC), ELAV-like RNA binding protein 1 (ELAVL1), Casitas B-lineage lymphoma-like 1 (CBLL1), and fragile X mental retardation protein 1 (FMR1)]. Previous studies have demonstrated that these regulators play pivotal roles in biological processes such as apoptosis, autophagy, and immune regulation, which are closely associated with inflammatory and immune-related diseases (16–18). Nevertheless, although recent studies have begun to explore the role of m^6^A modifications in sepsis (19, 20), the underlying molecular mechanisms remain poorly understood and should be elucidated.

**Figure 1:**
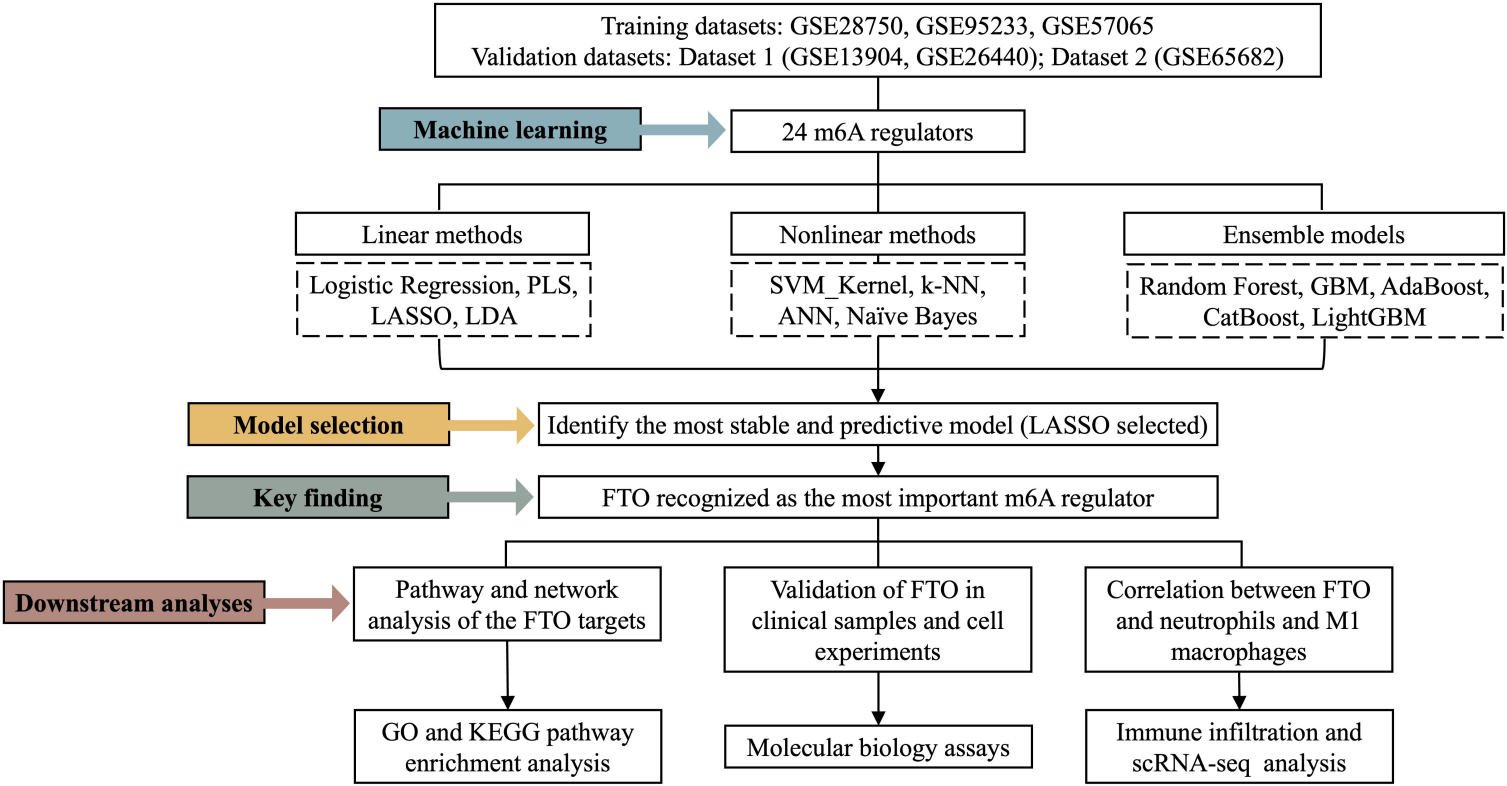
Diagram of the study.

In this study, we integrated transcriptomic data from the GSE13904, GSE26440, GSE28750, GSE57065, GSE65682, and GSE95233 datasets and systematically analysed 24 well-characterized m^6^A regulators. By constructing and comparing 14 supervised machine learning models, we evaluated the potential of these regulators as diagnostic biomarkers for sepsis. Furthermore, we investigated their downstream target genes and signalling pathways to elucidate the molecular mechanisms through which m^6^A regulators may contribute to the pathogenesis of sepsis (**Figure 1**). Collectively, the results of this study not only provide new evidence for the functional roles of m^6^A regulators in sepsis but also offer a theoretical basis for their potential application as diagnostic and therapeutic targets.

## 2 Materials and methods

### 2.1 Dataset collection and processing

A total of 661 samples were retrieved from the Gene Expression Omnibus (GEO) database, including 153 from healthy controls and 448 from patients with sepsis. The GSE13904, GSE26440, GSE28750, GSE95233, and GSE57065 datasets were obtained from the Affymetrix GPL570 platform (Affymetrix Human Genome U133 Plus 2.0 Array), while the GSE65682 dataset was derived from the Affymetrix GPL13667 platform (Affymetrix Human Genome U219 Array). Raw Affymetrix data were preprocessed using the robust multiarray averaging (RMA) algorithm implemented in the affy R package. A training set was constructed from GSE28750, GSE95233, and GSE57065 (GPL570 platform), and validation set 1 was generated by merging GSE13904 and GSE26440. Validation set 2 was constructed from GSE65682 (GPL13667 platform). All included samples were derived from peripheral blood.

To examine the expression profile of FTO across immune cell populations, single-cell RNA sequencing (scRNA-seq) data from the GSE249975 dataset were analysed.

### 2.2 Machine learning models

To systematically identify molecular signatures associated with sepsis, we employed a broad panel of machine learning algorithms covering linear, nonlinear, and ensemble learning paradigms. The linear methods included logistic regression, partial least squares (PLS) regression, least absolute shrinkage and selection operator (LASSO), and linear discriminant analysis (LDA). The nonlinear methods included support vector machines (SVMs) with a kernel function, k-nearest neighbour (k-NN) models, artificial neural networks (ANNs), and naïve Bayes methods. The ensemble approaches included random forest models, the generalized boosting method, the gradient boosting machine (GBM), adaptive boosting (AdaBoost), categorical boosting (CatBoost), and the light gradient boosting machine (LightGBM). Both the GBM and generalized boosting method were evaluated, as they represent widely used but technically distinct implementations of gradient boosting in biomedical research.

Model performance was assessed using receiver operating characteristic (ROC) curves, precision–recall (PR) curves, and the area under the curve (AUC). Moreover, sensitivity, specificity, and calibration were evaluated. The AUC was considered the primary metric. All analyses were conducted in R (version 4.3.2). The caret package was used for model training and cross-validation, while pROC, kernelshap, rmda, catboost, lightgbm, and DALEX were applied for model evaluation, interpretation, and visualization.

### 2.3 Pathway analysis

m6A2Target (http://m6a2target.canceromics.org/) is a comprehensive database that provides information on the target genes of writers, erasers, and readers (WERs). It integrates high-confidence targets validated by low-throughput experiments as well as potential targets supported by binding evidence from high-throughput sequencing or inferred from WER perturbation followed by high-throughput assays (21). The putative gene targets of key m^6^A regulators associated with disease diagnosis were identified using m6A2Target. Functional annotation of biological processes (BPs) was conducted using the GOplot package, while functional enrichment analyses were performed with the ClusterProfiler package (version 4.10.1), a widely used tool for the interpretation of omics data.

### 2.4 scRNA-seq analysis

Single-cell RNA sequencing data were obtained from a publicly available dataset derived from peritoneal immune cells of CLP-induced septic mice. The dataset was analyzed as provided by the original study. Raw 10× Genomics data were imported and converted into Seurat objects using the Seurat package (version 4.4.0). Quality control, normalization, dimensionality reduction, and clustering of peritoneal cell populations were subsequently performed according to standard Seurat workflows. Cell subsets were annotated based on reference transcriptomic datasets provided by the celldex package (version 1.12.0).

### 2.5 Clinical sample collection

In total, 12 patients with sepsis and 12 healthy controls were included. The diagnosis of sepsis was established according to the Third International Consensus Definitions for Sepsis and Sepsis Shock (Sepsis-3) (22). After receiving written informed consent, peripheral blood samples were collected and PBMCs and neutrophils were promptly separated. The experiment has been approved by the Ethics Committee of China-Japan Friendship Hospital (approval number 2024-KY-417-1).

### 2.6 Cecal ligation and puncture (CLP)–induced sepsis model

Polymicrobial sepsis was established in mice using the CLP procedure, as previously described (23) with minor modifications. Mice were anesthetized with 1.5% isoflurane delivered at 2 L/min. Following standard abdominal disinfection, a midline incision of approximately 1.5–2 cm was made to access the peritoneal cavity. The cecum was carefully isolated from the mesentery, and the distal portion was ligated with sterile silk at approximately halfway along its length. Two punctures were performed through the ligated portion using a 21-gauge needle, and a small amount of fecal material was gently extruded to ensure patency. The abdominal wall and skin were then closed in layers. Sham-operated controls underwent the same procedure except that the cecum was neither ligated nor punctured.

### 2.7 Cell culture and treatments

RAW264.7 cells were chosen for macrophage-related experiments. To suppress the expression of FTO, Fto-specific siRNA (siFTO) and negative control siRNA (siNC) were chemically synthesized by D-Nano Therapeutics (China). Transfection was carried out using the CALNP™ RNAi transfection reagent (D-Nano Therapeutics, China) following the manufacturer’s protocol. For rescue assays, transfected cells were treated with 20 µM PI3K agonist 740 Y-P (HY-P0175, MCE). The siRNA sequences are listed in **Supplementary Table 1**.

Primary neutrophils were isolated from mouse bone marrow as previously described and cultured in RPMI-1640 with 10% FBS (24). Cells were stimulated with the FTO inhibitor FB23-2 (HY127103, MCE) and/or 10 µM PI3K inhibitor LY294002 (HY10108, MCE) prior to downstream assays.

### 2.8 Real-time qPCR analysis and Western blot analysis

RNA isolation and real-time quantitative polymerase chain reaction (RT–qPCR) analysis were performed as previously described (25). GAPDH was used as the internal control. The primer sequences employed in this study are listed in **Supplementary Table 2**.

Protein extraction and Western blot analysis were performed as previously described (25). The primary antibody used was anti-FTO (EPR24440-12, 1:50 dilution; abcam), p-PI3K (T40116M, 1:1000; Abmart), PI3K (T40115M, 1:1000; Abmart), p-AKT (#4060, 1:1000; CST), AKT (#4685, 1:1000; CST), with GAPDH and β-actin serving as the internal control. Band intensities on autoradiograms were quantified by densitometric analysis using Quantity One software (Bio-Rad).

### 2.9 Flow cytometric analysis and enzyme linked immunosorbent assay

Following stimulation with LPS (200 ng/mL) for 24 h, the cells were harvested and washed with PBS. Surface staining was performed at 4 °C for 30 min using PE-conjugated anti-F4/80 (cat. 123109), FITC-conjugated anti-CD11b (cat. 101205), and Brilliant Violet 421-conjugated anti-CD86 (cat. 105031). Flow cytometry was conducted on a Beckman instrument, and the data were analysed using FACSDeva software.

After transfection and stimulation, the cell culture supernatants were collected, and the level of TNF-α was quantified using a mouse TNF-α ELISA kit (E-HSEL-M0009; Elabscience, China) according to the manufacturer’s protocol.

### 2.10 Intracellular reactive oxygen species (ROS) detection

Intracellular reactive oxygen species (ROS) detection generation was assessed using DCFH-DA (Reactive Oxygen Species Assay Kit, Solarbio, cat. CA1410) according to the manufacturer’s protocol. Following treatment, cells were incubated with 1:750 diluted DCFH-DA for 30 min at 37 °C in the dark, washed twice with serum-free medium, and counterstained with Hoechst 33342 (Solarbio). Fluorescence was recorded at 488/525 nm (excitation/emission), and ROS levels were expressed as mean fluorescence intensity.

### 2.11 Immunofluorescence staining

Immunofluorescence staining was performed to evaluate FTO expression and its spatial association with immune cell populations in intestinal tissues. Tissue sections were fixed with 4% paraformaldehyde for 20 min, permeabilized with 0.3% Triton X-100 for 10 min, and blocked with 5% bovine serum albumin for 1 h at room temperature. Sections were then incubated overnight at 4 °C with primary antibodies against FTO (EPR24440-12, 1:50 dilution; abcam), F4/80 (ab04467, 1:200 dilution; abcam) or Ly6G (cat. 127609, 1:200 dilution; BioLegend). After washing, sections were incubated with Alexa Fluor 488- or 594-conjugated secondary antibodies (1:500) for 1 h at room temperature in the dark, followed by nuclear counterstaining with DAPI. Images were acquired using a Zeiss laser scanning confocal microscope under identical settings for all groups.

### 2.12 Statistical analyses

Statistical analyses were performed using GraphPad Prism (GraphPad Software, San Diego, CA) and R software (version 4.3.2). Comparisons between two groups were conducted using Student’s t-test. For experiments involving more than two groups, one-way analysis of variance (ANOVA) followed by Tukey’s *post hoc* multiple-comparison test was applied. Data are presented as the mean ± standard error of the mean (SEM), and P < 0.05 was considered statistically significant.

## 3 Results

### 3.1 Performance of sepsis classification approaches using m^6^A regulators

**Figure 2:**
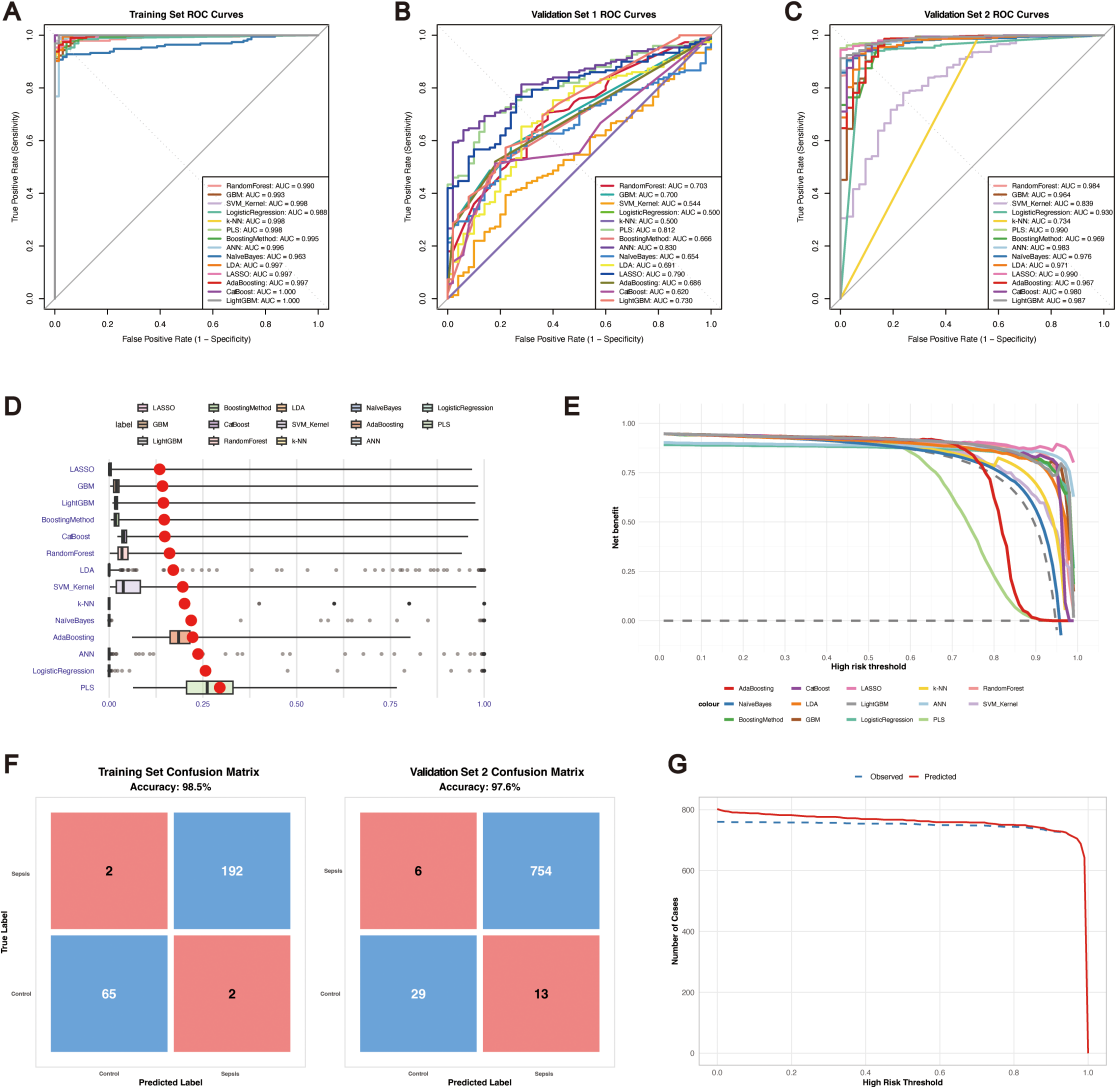
Performance of machine learning models for sepsis diagnosis based on m^6^A regulators. **(A)** ROC curves of 14 machine learning models for the training set. **(B)** ROC curves for validation set 1. **(C)** ROC curves for validation set 2. **(D)** Residual boxplots of the model predictions. **(E)** Decision curve analysis (DCA) of all the models. **(F)** Confusion matrices of the LASSO model for the training set and validation set 2. **(G)** Clinical impact curves (CICs) of the LASSO model for the training and validation set 2 cohorts.

Given the pivotal role of m^6^A methylation regulators in shaping inflammation and immunity, we systematically evaluated the diagnostic potential of 24 m^6^A regulators for sepsis using publicly available datasets. On the basis of the expression profiles of these regulators, we constructed diagnostic models employing 14 distinct machine learning algorithms, namely, logistic regression, PLS, LASSO, LDA, SVM_Kernel, k-NN, ANN, naïve Bayes, random forest, generalized boosting, GBM, AdaBoost, CatBoost, and LightGBM algorithms. Rigorous cross-validation was conducted on the basis of the training set and two independent validation cohorts. The cross-validation performance in the training set is summarized in **Supplementary Table 3**, with all the models achieving AUC values greater than 0.9 (**Figure 2A**). To further place these diagnostic findings into a broader regulatory context, we conducted integrative pathway-level analyses across multiple independent sepsis transcriptomic datasets. Using ssGSEA, we performed a systematic comparison of m^6^A RNA modification versus other RNA modification and epigenetic regulatory pathways, including m1A, m5C, and m7G RNA modifications, as well as DNA methylation. Notably, m6A-related regulatory pathways exhibited the most consistent and pronounced dysregulation between control and sepsis samples across datasets (**Supplementary Figure 1**), thereby prioritizing m^6^A modification as a key regulatory mechanism for subsequent mechanistic investigation in this study.

To compare the diagnostic performance across algorithms, we next assessed their classification accuracy in the validation datasets. In validation set 1 (**Figure 2B**), the ANN model achieved the highest AUC (0.83), while logistic regression and k-NN had the lowest AUC values (~0.50). However, the specificity in validation set 1 was nearly zero (**Supplementary Table 4**), suggesting potential overdiagnosis; therefore, subsequent analyses focused mainly on validation set 2. In validation set 2 (**Figure 2C**), all the models achieved AUC values greater than 0.70, with LASSO reaching the highest AUC (0.99). Models including the GBM, logistic regression, PLS, generalized boosting, ANN, LDA, LASSO, AdaBoost, and LightGBM models maintained balanced accuracy, sensitivity, and specificity above 0.60, indicating satisfactory generalizability (**Table 1**).

**Table 1:** Model performance of the 14 classifiers for validation set 2.

Models	Sensitivity	Specificity	Accuracy	PPV	NPV
Random Forest	0.979381443298969	0.940298507462687	0.969348659003831	0.979381443298969	0.940298507462686
GBM	0.984536082474227	0.91044776119403	0.96551724137931	0.969543147208122	0.953125
SVM_Kernel	0.984536082474227	0.985074626865672	0.984674329501916	0.994791666666667	0.956521739130435
Logistic Regression	0.958762886597938	0.925373134328358	0.950191570881226	0.973821989528796	0.885714285714286
k-NN	0.984536082474227	0.970149253731343	0.980842911877395	0.989637305699482	0.955882352941177
PLS	0.979381443298969	0.970149253731343	0.977011494252874	0.989583333333333	0.942028985507246
Generalized Boosting Method	0.979381443298969	0.940298507462687	0.969348659003831	0.979381443298969	0.940298507462686
ANN	0.984536082474227	0.985074626865672	0.984674329501916	0.994791666666667	0.956521739130435
Naïve Bayes	0.93298969072165	0.82089552238806	0.904214559386973	0.937823834196891	0.808823529411765
LDA	0.969072164948454	0.970149253731343	0.969348659003831	0.989473684210526	0.915492957746479
LASSO	0.984536082474227	0.940298507462687	0.973180076628352	0.979487179487179	0.954545454545455
AdaBoosting	0.989690721649485	0.940298507462687	0.977011494252874	0.979591836734694	0.969230769230769
CatBoost	1	0.985074626865672	0.996168582375479	0.994871794871795	1
LightGBM	1	0.985074626865672	0.996168582375479	0.994871794871795	1

In the residual boxplots (**Figure 2D**), compared with the other models, the LASSO, GBM, LightGBM, and generalized boosting models resulted in smaller residuals, indicating better generalization performance. Conversely, models such as PLS, logistic regression and ANN showed higher residuals, suggesting potential overfitting and limited robustness on unseen data. Decision curve analysis (**Figure 2E**) further demonstrated that the LASSO model consistently provided greater net clinical benefit than the other models across threshold probabilities between 0.5 and 1. Confusion matrices (**Figure 2F**) confirmed the robustness of the LASSO model, with accuracies of 98.5% in the training set and 97.6% in validation set 2. According to the clinical impact curves (**Figure 2G**), the LASSO model maintained stable predictive performance across both the training and validation cohorts. Collectively, these results highlight that, particularly for validation set 2, the LASSO model has strong potential for clinical application in the blood-based diagnosis of sepsis.

### 3.2 Important m^6^A methylation regulators in the sepsis classification

**Figure 3:**
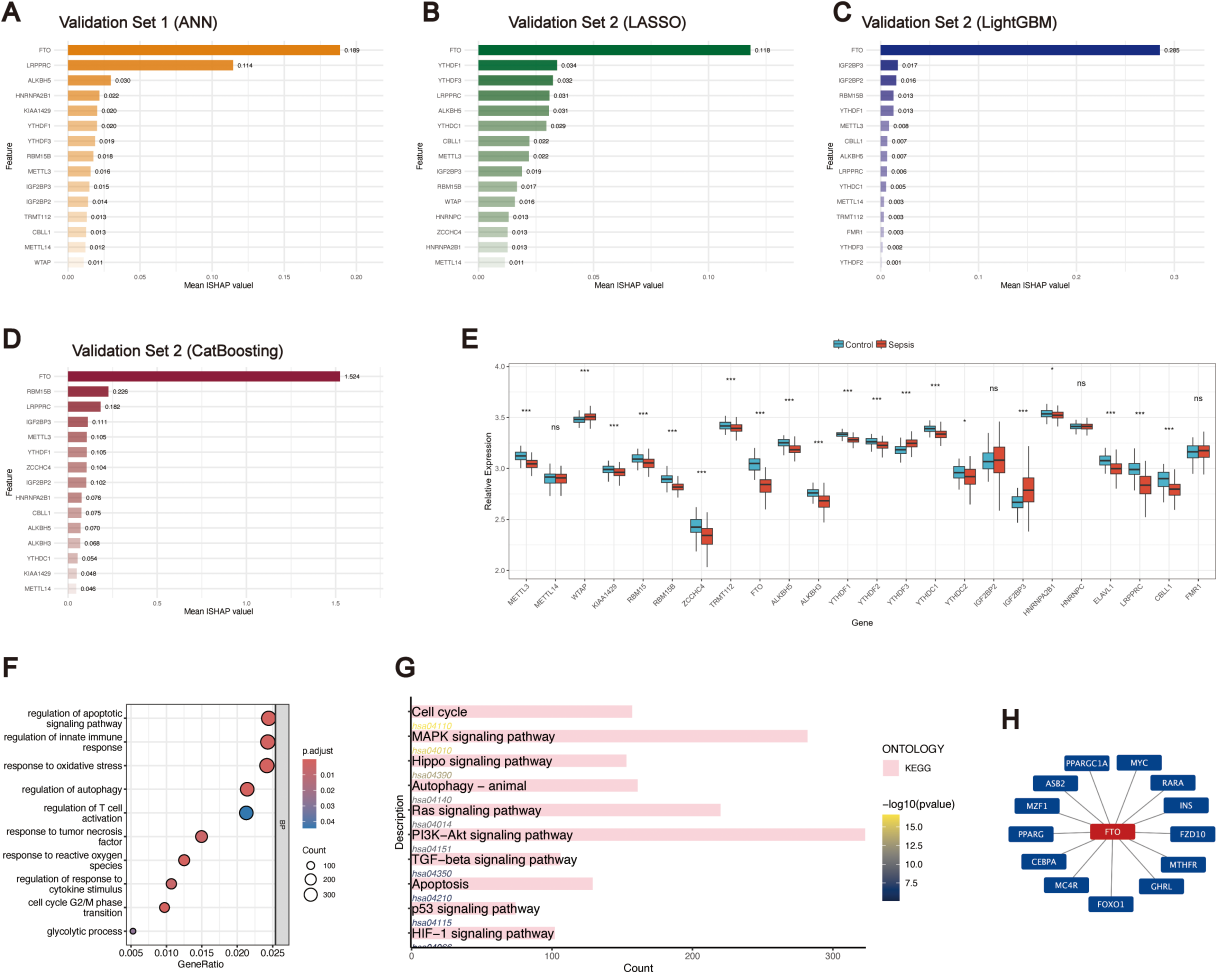
Identification of FTO as a key m^6^A regulator in sepsis and its functional enrichment analysis. **(A–D)** Feature importance rankings of 24 m^6^A regulators across different machine learning models: **(A)** ANN in validation set 1; **(B)** LASSO model in validation set 2; **(C)** LightGBM in validation set 2; **(D)** CatBoost model in validation set 2. **(E)** Differential expression of 24 m^6^A regulators between sepsis patients and healthy controls. Boxplots show the median and interquartile range; ***P < 0.001, **P < 0.01, *P < 0.05, ns = not significant. **(F)** BP enrichment analysis of FTO-predicted target genes. **(G)** KEGG pathway enrichment analysis of FTO-predicted target genes. **(H)** Cytoscape-based visualization of the FTO–target gene interaction network.

Using these machine learning approaches, different candidate biomarkers were identified across the validation sets. Given that the optimal models differed between the two validation cohorts, we calculated the mean |SHAP| values of 24 m^6^A regulators to evaluate their relative contributions to model construction. In validation set 1, the ANN model highlighted FTO as the top contributor, with a mean |SHAP| value of 0.189 (**Figure 3A**). In validation set 2, we focused on three models with superior diagnostic performance—LASSO, LightGBM, and CatBoost—all of which consistently ranked FTO as the most important feature, with mean |SHAP| values of 0.118 (**Figure 3B**), 0.285 (**Figure 3C**), and 1.524 (**Figure 3D**), respectively. Furthermore, analysis of the training set revealed significant differential expression of several m^6^A regulators between sepsis patients and healthy controls, among which FTO displayed one of the most pronounced differences (**Figure 3E**). Collectively, these findings underscore the central role of FTO in diagnostic modelling for sepsis.

### 3.3 Pathway and network analysis of the FTO targets

To further explore the potential roles of m^6^A regulators in sepsis and to investigate the pathways involved, we used the m6A2Target database to predict downstream targets. For FTO, 105 experimentally validated targets and 23,943 predicted targets were identified (**Supplementary Table 5**). On the basis of the predicted targets, BPs enrichment analysis was performed using the ClusterProfiler package, revealing that FTO-related genes are involved mainly in apoptosis, autophagy, oxidative stress, innate immunity, and cell cycle regulation, all of which are critically involved in the pathogenesis of sepsis (**Figure 3F**). KEGG pathway enrichment analysis further demonstrated that the predicted FTO targets were significantly enriched in key signalling pathways, including the PI3K-Akt signalling pathway, MAPK signalling pathway, Ras signalling pathway, cell cycle, Hippo signalling pathway, autophagy, TGF-beta signalling pathway, apoptosis, p53 signalling pathway, and HIF-1 signalling pathway (**Figure 3G**). To better illustrate the regulatory relationships, a Cytoscape-based network was constructed, which revealed that FTO was closely connected with several hub targets, such as MC4R, ASB2, MYC, PPARG, and FOXO1 (**Figure 3H**).

### 3.4 Immune landscape and single-cell characterization of FTO in sepsis

**Figure 4:**
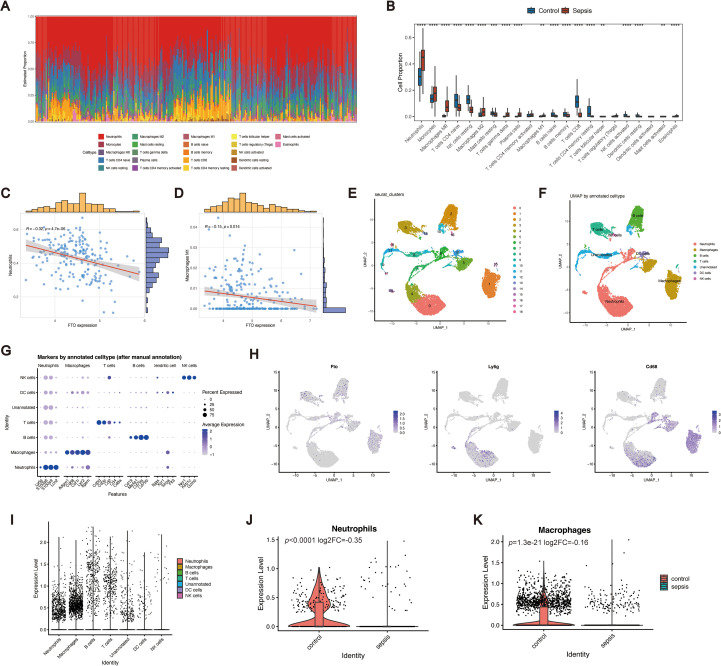
Immune landscape and single-cell characterization of FTO in sepsis. **(A)** Immune cell composition estimated by CIBERSORT in the training dataset. **(B)** Comparison of immune cell proportions between healthy controls (HC) and sepsis patients. **(C)** Correlation between the proportion of neutrophils and FTO expression. **(D)** Correlation between the M1 macrophage proportion and FTO expression. **(E)** UMAP plot showing 18 distinct clusters. **(F)** Cell type annotation of six major immune cell populations using SingleR. **(G)** Dot plot of canonical marker genes used for cell type annotation. **(H)** UMAP feature plots showing the expression of FTO, Ly6g, and Cd68. **(I)** Distribution of FTO expression across different immune cell subsets. **(J, K)** Violin plots comparing FTO expression levels in neutrophils **(J)** and macrophages **(K)** between the control and sepsis groups.

Accumulating evidence has demonstrated that m^6^A modification serves as a critical regulatory factor in immune responses and inflammatory networks. To elucidate the immunological features associated with sepsis and FTO expression, we first evaluated the immune scores and immune cell fractions. In the training cohort, neutrophils accounted for the greatest proportion of infiltrating cells, followed by monocytes and M0 macrophages (**Figure 4A**). Comparative analysis revealed significant differences in the proportions of multiple immune cell subsets, including neutrophils, monocytes, M0 macrophages, CD4+ naive T cells, resting NK cells, M2 macrophages, M1 macrophages, resting mast cells, gamma delta T cells, plasma cells, naive B cells, memory B cells, CD8+ T cells, resting CD4+ memory T cells, follicular helper T cells, activated resting NK cells, resting dendritic cells, activated mast cells, and eosinophils (**Figure 4B**), between sepsis patients and healthy controls. Among these subsets, neutrophils and M1 macrophages are of particular interest because of their high relevance to sepsis pathophysiology (26–28). The proportion of neutrophils was significantly greater in sepsis patients than in controls and was negatively correlated with FTO expression (**Figure 4C**). Similarly, the proportion of M1 macrophages was markedly greater in sepsis patients and correlated with FTO levels (**Figure 4D**), suggesting the potential involvement of FTO in M1 macrophage activation.

To further characterize the relationship between FTO expression and neutrophil/macrophage populations, we performed scRNA-seq analysis using the GSE249975 dataset. Uniform manifold approximation and projection (UMAP) revealed 18 distinct cellular clusters (**Figure 4E**). On the basis of SingleR annotation, six major immune cell types were defined: neutrophils, macrophages, B cells, T cells, dendritic cells (DCs), and natural killer (NK) cells (**Figures 4F, G**). Notably, FTO was coexpressed with canonical neutrophil (Ly6g) and macrophage (Cd68) markers (**Figure 4H**). Cell type-specific analysis further revealed that FTO was predominantly expressed in neutrophils and macrophages (**Figure 4I**). When FTO expression was compared between the control and sepsis groups, we observed significant downregulation of FTO in both neutrophils and macrophages from sepsis patients (**Figures 4J, K**). These findings highlight the close association of FTO with innate immune cells and suggest its potential involvement in the dysregulated immune responses characteristic of sepsis.

### 3.5 Functional validation of FTO in macrophages during sepsis

**Figure 5:**
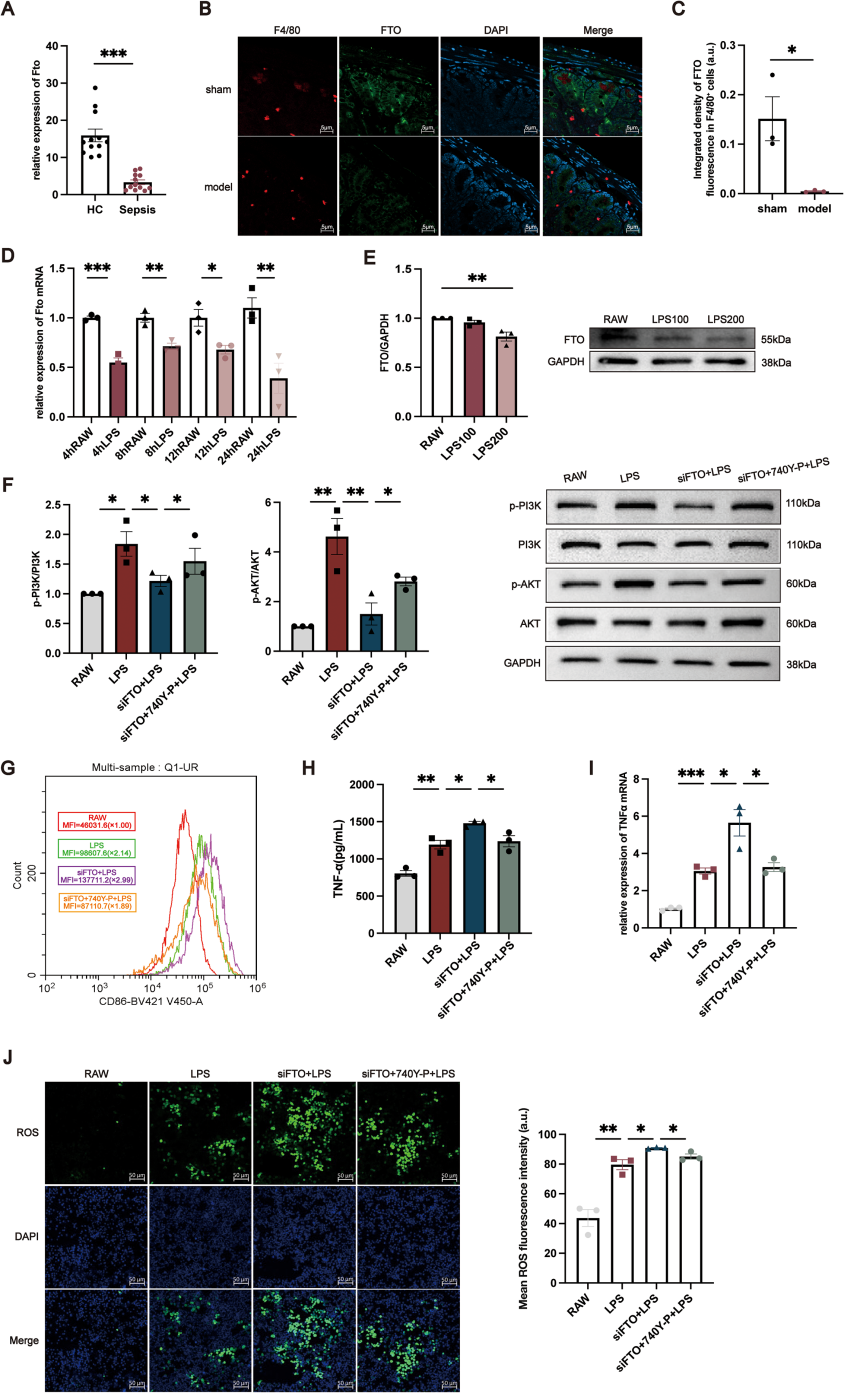
Functional validation of FTO in macrophages during sepsis. **(A)** Relative FTO mRNA expression in PBMCs from HCs and sepsis patients. **(B)** Representative immunofluorescence images showing the localization of FTO in intestinal tissues from sham-operated and CLP mice. FTO signals partially overlap with the macrophage marker F4/80. F4/80 is shown in red, FTO in green, and nuclei are counterstained with DAPI (blue). **(C)** Quantitative analysis of the integrated fluorescence intensity of FTO within F4/80^+^ macrophages in intestinal tissues from sham and CLP mice. **(D)** Time-dependent changes in FTO mRNA expression in RAW264.7 cells following LPS stimulation for 4, 8, 12, and 24 (h) **(E)** Western blot analysis of FTO protein expression in RAW264.7 cells treated with LPS (100 or 200 ng/mL) for 24 (h) For panels **(F–J)**, RAW264.7 cells were divided into four groups: untreated control (RAW), LPS-treated (LPS), siFTO-transfected (LPS + siFTO), and siFTO-transfected cells treated with the PI3K agonist 740 Y-P (LPS + siFTO + 740 Y-P). **(F)** Western blot analysis of phosphorylated PI3K (p-PI3K) and total PI3K, both representing the PI3K p110 catalytic subunit (apparent MW ~110 kDa), phosphorylated AKT (p-AKT, ~60 kDa), total AKT (~60 kDa), and GAPDH (~38 kDa) in RAW264.7 cells. **(G)** Flow cytometric analysis of CD86 expression in RAW264.7 cells, presented as MFI. **(H, I)** TNF-α protein levels **(H)** and mRNA expression **(I)** in RAW264.7 cells under the indicated conditions. **(J)** Representative images of intracellular ROS fluorescence in RAW264.7 cells and quantitative analysis of mean fluorescence intensity (a.u.). The data are presented as the mean ± SEM. *P < 0.05, **P < 0.01, ***P < 0.001.

To validate the clinical relevance of FTO dysregulation, we first examined its expression in PBMCs isolated from sepsis patients and healthy controls. RT–qPCR analysis demonstrated that FTO mRNA levels were significantly reduced in PBMCs from sepsis patients compared with those from healthy individuals (**Figure 5A**). We next investigated FTO expression in intestinal tissue from mice subjected to CLP–induced polymicrobial sepsis and sham-operated controls. Immunofluorescence staining revealed that FTO signals partially colocalized with F4/80^+^ macrophages in the intestinal mucosa, indicating that FTO is expressed in intestinal macrophage populations (**Figure 5B**). To further assess alterations in macrophage-associated FTO expression under septic conditions, the integrated fluorescence intensity of FTO within F4/80^+^ macrophages was quantitatively analyzed. As shown in **Figure 5C**, FTO in F4/80^+^ macrophages was significantly decreased in CLP mice compared with sham controls. Consistent with the *in vivo* findings, *in vitro* stimulation of RAW264.7 macrophages with LPS resulted in a marked downregulation of FTO mRNA expression at 4, 8, 12, and 24 h (**Figure 5D**). A corresponding reduction in FTO protein levels was observed after 24 h of LPS treatment (**Figure 5E**). To determine the functional role of FTO in macrophage activation, FTO expression was silenced in RAW264.7 cells using specific siRNAs. The knockdown efficiency was confirmed by RT–qPCR and Western blotting (**Supplementary Figure 2A, B**). To dissect the molecular mechanism by which FTO modulates macrophage activation, we focused on the PI3K/AKT signaling pathway based on KEGG pathway enrichment analysis. Western blot results showed that while LPS stimulation induced PI3K and AKT phosphorylation, siFTO significantly suppressed the levels of p-PI3K and p-AKT compared to the LPS control group (**Figure 5F**). This suggests that FTO is required for the sustainment of PI3K/AKT signaling during inflammatory responses. To determining whether the inhibition of PI3K/AKT signaling is responsible for the exacerbated inflammatory phenotype observed in FTO-deficient macrophages, we performed a rescue experiment using a specific PI3K agonist. RAW264.7 cells were transfected with siFTO and subsequently treated with LPS in the presence or absence of the PI3K agonist. As expected, the PI3K agonist effectively restored the phosphorylation levels of PI3K and AKT in siFTO-transfected cells (**Figure 5F**). We then evaluated the inflammatory phenotype. Flow cytometric analysis revealed that FTO silencing significantly increased the mean fluorescence intensity of the M1 marker CD86 compared with LPS treatment alone. Notably, treatment with the PI3K agonist significantly attenuated this siFTO-induced upregulation of CD86 (**Figure 5G**). Consistently, RT–qPCR and ELISA analyses demonstrated that the elevated TNF-α mRNA expression and protein secretion caused by FTO knockdown were markedly reversed by the restoration of PI3K/AKT signaling (**Figures 5H, I**). Furthermore, the enhanced intracellular ROS production observed in the siFTO group was significantly mitigated by the PI3K agonist (**Figure 5J**). Collectively, these data provide rigorous evidence that FTO prevents excessive macrophage inflammation, at least in part, by maintaining PI3K/AKT signaling axis.

### 3.6 Functional validation of FTO in neutrophils during sepsis

**Figure 6:**
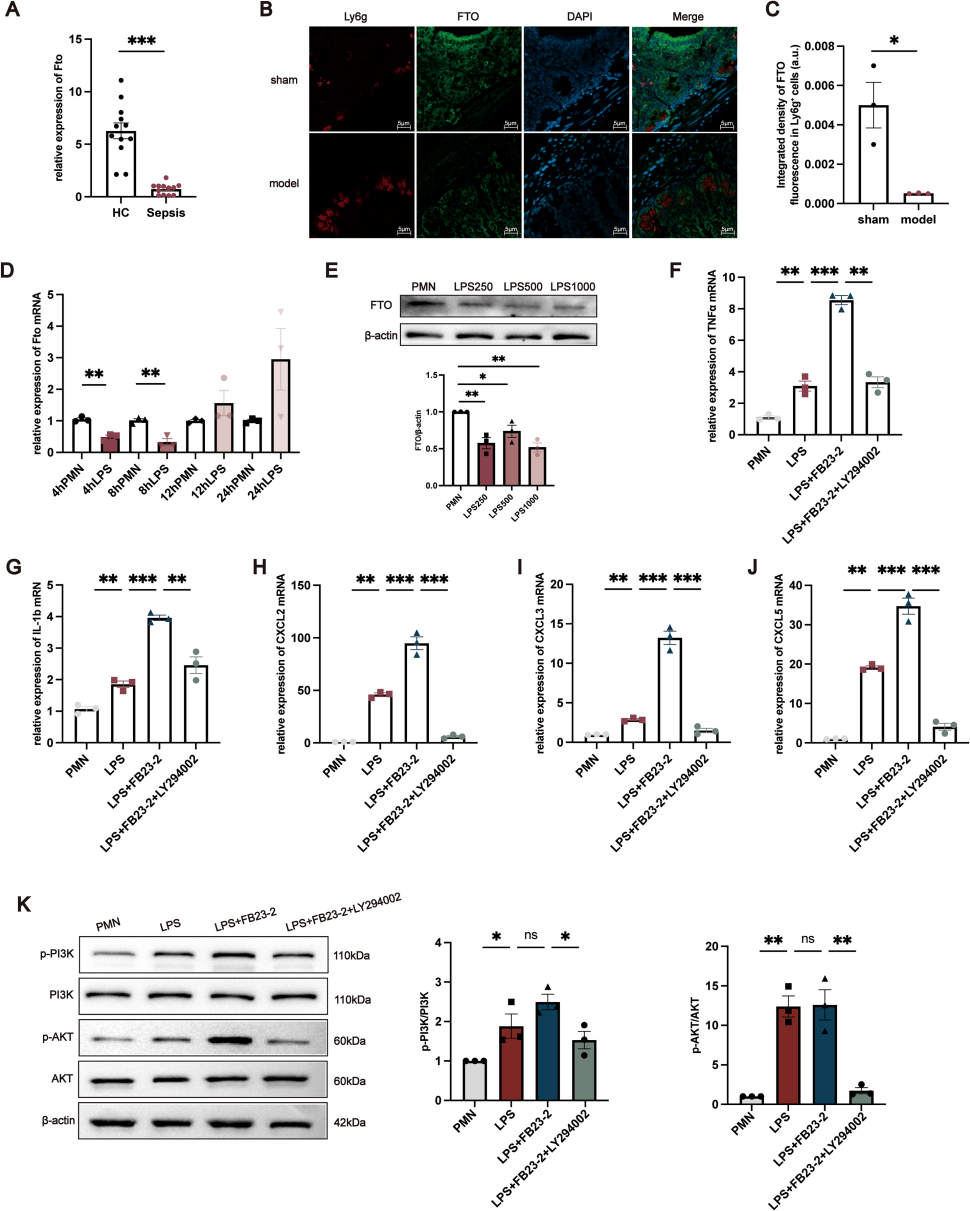
Functional validation of FTO in neutrophils during sepsis. **(A)** Relative FTO mRNA expression in neutrophils from HCs and sepsis patients. **(B)** Representative immunofluorescence images showing the localization of FTO in intestinal tissues from sham-operated and CLP mice. FTO signals partially overlap with the neutrophil marker Ly6G. Ly6G is shown in red, FTO in green, and nuclei are counterstained with DAPI (blue). **(C)** Quantitative analysis of the integrated fluorescence intensity of FTO within Ly6G^+^ neutrophils in intestinal tissues from sham and CLP mice. **(D)** Time-dependent changes in FTO mRNA expression in neutrophils following LPS stimulation for 4, 8, 12, and 24 (h) **(E)** Western blot analysis of FTO protein expression in neutrophils treated with LPS (250, 500 or 1000 ng/mL) for 8 (h) For panels **(F–K)**, neutrophils were divided into four groups: untreated control, LPS-treated, FB23-2–treated, and FB23-2 + LY294002–treated cells. **(F–J)** Relative mRNA expression levels of proinflammatory mediators (TNF-α, IL-1β, CXCL2, CXCL3, and CXCL5) in neutrophils under the indicated conditions. **(K)** Western blot analysis of phosphorylated PI3K (p-PI3K) and total PI3K, both representing the PI3K p110 catalytic subunit (apparent MW ~110 kDa), phosphorylated AKT (p-AKT, ~60 kDa), total AKT (~60 kDa), and bei’taβ-actin (~42 kDa) in neutrophils under the indicated conditions. The data are presented as the mean ± SEM. *P < 0.05, **P < 0.01, ***P < 0.001.

To validate the clinical relevance of FTO dysregulation in neutrophils, we first examined its expression in neutrophils isolated from sepsis patients and healthy controls. RT–qPCR analysis revealed that FTO mRNA levels were significantly reduced in neutrophils from sepsis patients compared with those from healthy individuals (**Figure 6A**). We next investigated FTO expression in intestinal tissues from mice subjected to CLP–induced polymicrobial sepsis and sham-operated controls. Immunofluorescence staining demonstrated that FTO signals partially colocalized with Ly6G^+^ neutrophils in the intestinal mucosa, indicating that FTO is expressed in intestinal neutrophils (**Figure 6B**). To further assess alterations in neutrophil-associated FTO expression under septic conditions, the integrated fluorescence intensity of FTO within Ly6G^+^ neutrophils was quantitatively analyzed. As shown in **Figure 6C**, FTO expression in Ly6G^+^ neutrophils was significantly decreased in CLP mice compared with sham controls. *In vitro*, LPS stimulation induced dynamic changes in FTO expression in neutrophils. FTO mRNA levels were markedly downregulated at 4 and 8 h, followed by a gradual increase at 12 and 24 h after LPS treatment (**Figure 6D**). Consistent changes in FTO protein expression were observed at 8 h (**Figure 6E**). To further explore the functional relevance of FTO in neutrophils, the selective FTO inhibitor FB23–2 was applied to suppress FTO activity. RT–qPCR analysis showed that FB23–2 treatment significantly increased the mRNA expression of proinflammatory mediators, including TNF-α, IL-1β, CXCL2, CXCL3, and CXCL5 (**Figures 6F–J**). Based on KEGG pathway enrichment analysis, the PI3K/AKT signaling pathway was further examined. Pharmacological inhibition of PI3K with LY294002 markedly reversed the FB23-2–induced upregulation of inflammatory gene expression (**Figure 6K**). Western blot analysis demonstrated that LPS stimulation increased the phosphorylation levels of PI3K and AKT in neutrophils compared with untreated controls. Notably, FB23–2 treatment further enhanced the phosphorylation of PI3K and AKT without significantly affecting their total protein levels, whereas PI3K inhibition restored their phosphorylation status (**Figure 6K**). These results suggest that FTO dysregulation in neutrophils is associated with altered PI3K/AKT signaling during sepsis.

## 4 Discussion

Sepsis is a life-threatening systemic inflammatory response syndrome triggered by a dysregulated immune response to infection, which can lead to multiple organ dysfunction and high mortality. Despite advancements in anti-infective therapies and organ support technologies, sepsis remains among the leading causes of death and long-term complications in intensive care units worldwide (29, 30). The pathogenesis of sepsis is complex and involves factors such as excessive inflammation, immune suppression, and metabolic imbalance. Accumulating evidence suggests that mutations or abnormal expression of genes encoding m^6^A methylation regulators are closely related to the occurrence of inflammation and immune-related diseases (31). Recent bioinformatics and experimental studies have shown that several m^6^A regulatory factors are closely associated with inflammation levels and immune cell infiltration in sepsis (32, 33). Therefore, a deeper understanding of the role of m^6^A methylation regulators in the development and progression of sepsis will not only help reveal its molecular pathological basis but also provide new insights for early diagnosis, prognosis assessment, and targeted therapy.

In these studies, on the basis of the expression profiles of m^6^A methylation regulators and machine learning methods, we constructed multiple binary predictive classification models to assess the performance of these factors in distinguishing clinical classifications. Among these models, the LASSO model demonstrated the most stable performance in the validation set, with a high AUC and low signs of overfitting. However, when applied to paediatric whole blood samples, the LASSO model did not show the same level of performance. Possible reasons for this include the physiological differences between paediatric and adult samples, the greater variability in the expression patterns of m^6^A regulators in children, or the small sample size leading to unstable signals or overfitting during training. Our main goal was not to immediately introduce a clinically applicable diagnostic tool but rather to explore the association between m^6^A regulatory factor expression and clinical classification. Through model evaluations, including cross-validation and independent validation set testing, we concluded that these m^6^A methylation regulators have a certain degree of classification accuracy, laying the foundation for the development of diagnostic tools and confirming their potential diagnostic value. Nevertheless, there are several limitations of this study. For example, we used only peripheral blood samples to establish and validate the classifiers. While peripheral blood is easily collected and highly reproducible, it may not fully reflect the local immune status in specific organs or infection sites. Future studies should assess the expression of m^6^A regulators in multiple tissue types to enhance the comprehensiveness of the model. Additionally, differences in gene expression regulation, pathological response, and immune reactions between adult and paediatric samples have yet to be fully explored, and the generalizability of the current model across different age groups has not been adequately tested. It will be necessary to validate the model in larger retrospective and prospective clinical cohorts covering different age groups in the future.

In recent years, machine learning–based approaches have been increasingly applied in biomedical research to integrate high-dimensional omics data in an unbiased manner and to identify disease-associated regulatory factors (34–36). In the present study, by combining m^6^A regulator expression profiles with multiple feature selection and modeling algorithms, including LASSO, LightGBM, and random forest, we identified a set of robust candidate m^6^A regulators associated with sepsis. The use of multiple algorithms improved the reproducibility of feature selection and reduced potential false-positive results arising from reliance on a single model (37). Among these candidates, FTO consistently ranked highest in variable importance across different models, demonstrating stable predictive performance and a strong association with sepsis.Previous studies have reported that FTO plays key roles in inflammation and immune regulation in sepsis (38–40). In an LPS-induced endotoxin shock model, targeting FTO attenuated the inflammatory response, indicating that FTO may be involved in regulating the inflammatory cascade (41). In another study, overexpression of FTO improved survival in septic mice and reduced LPS-induced cardiac dysfunction, with targeting of the FTO/BACH1 axis showing therapeutic potential for sepsis-induced cardiac injury (42). Moreover, FTO has recently been identified as a cardiac protective factor (43). These findings provide strong biological support for our identification of FTO as a key m^6^A regulatory factor using machine learning. Further functional enrichment analysis revealed that FTO is involved in processes such as apoptosis, autophagy, oxidative stress, and cell cycle regulation. Its potential regulatory pathways include the PI3K/Akt, MAPK, and Ras signalling pathways, all of which are frequently reported in the development of sepsis (44, 45). Based on these results, we focused on the PI3K/AKT pathway as a candidate downstream signaling axis for experimental validation. In RAW264.7 macrophages, genetic silencing of FTO under LPS stimulation markedly suppressed PI3K and AKT phosphorylation and was accompanied by enhanced inflammatory responses. Conversely, FTO overexpression restored PI3K/AKT activation and effectively reversed the pro-inflammatory phenotype, providing direct rescue evidence at both the signaling and functional levels. However, the precise causal hierarchy between PI3K/AKT activation and downstream inflammatory mediators warrants further investigation. These findings indicate that FTO is required to maintain PI3K/AKT pathway activation in macrophages, where this pathway functions as a protective, anti-inflammatory regulatory axis during septic inflammation.

Given the prominent role of neutrophils in sepsis-associated immune dysregulation, we further explored whether FTO-dependent regulation of PI3K/AKT signaling is conserved across immune cell types. Immune infiltration analysis revealed that neutrophils exhibited the most pronounced alterations in sepsis, consistent with previous reports showing marked changes in neutrophil abundance, maturation status, and effector functions during early disease stages (26, 46). Neutrophils and macrophages are two major innate immune cell populations critically involved in sepsis, exerting distinct yet complementary roles in inflammatory initiation and immune regulation (47, 48). In neutrophil cells, pharmacological inhibition of FTO using FB23–2 enhanced inflammatory and chemotactic gene expression and was associated with sustained activation of the PI3K/AKT pathway. Importantly, co-treatment with the PI3K inhibitor LY294002 significantly attenuated both PI3K/AKT phosphorylation and the associated inflammatory phenotypes, indicating a pathway-dependent rescue effect in this cellular context. These results highlight a cell-type–dependent regulatory pattern in which PI3K/AKT signaling exerts distinct functional roles in macrophages and neutrophils during sepsis. Consistent with these observations, our analyses in PBMCs and isolated neutrophils from sepsis patients demonstrated generally reduced FTO expression. At the cellular level, FTO expression in neutrophils was transiently suppressed at early time points following LPS stimulation, with partial recovery at later stages, whereas FTO expression in M1 macrophages remained persistently low. Moreover, siFTO interference significantly upregulated CD86 expression, supporting an anti-inflammatory role of FTO in restraining macrophage inflammatory polarization. Previous studies have shown that FTO regulates macrophage inflammation through modulation of SOCS1 mRNA stability and inhibition of ferroptosis (49–51), and recent evidence suggests that post-translational modifications such as O-GlcNAcylation may further influence FTO stability and inflammatory feedback control (52). In contrast, the mechanistic role of FTO in neutrophil activation remains largely unexplored. In summary, our study integrates machine learning–based discovery with cell-type–specific functional validation to identify FTO as a key m^6^A demethylase involved in sepsis progression. Our findings suggest that FTO modulates inflammatory responses in macrophages and neutrophils, at least in part, through PI3K/AKT-dependent mechanisms, while the precise FTO-regulated targets and methylation events underlying these effects warrant further investigation.

The diagnostic model for sepsis, established on the basis of public databases, demonstrated high predictive performance in the independent validation set, indicating its potential clinical application value. However, before this model can be applied in clinical practice, further validation of its robustness and generalizability in larger sample sizes and multicentre cohorts is necessary. In addition, at the level of functional validation, the present study primarily employed loss-of-function approaches to demonstrate the involvement of FTO in regulating macrophage polarization and neutrophil function. Although these findings support the requirement of endogenous FTO activity, complementary gain-of-function evidence is currently lacking. Future studies will therefore focus on establishing FTO overexpression models *in vitro* and on generating adeno-associated virus (AAV)–mediated FTO overexpression animal models *in vivo*, which will allow a more systematic evaluation of the dosage-dependent and cell-specific causal roles of FTO in macrophages and neutrophils. From a mechanistic perspective, the bioinformatic analyses in this study were intended to characterize the global regulatory landscape associated with FTO rather than to resolve causal relationships for individual downstream targets. Our results indicate that FTO is linked to the regulation of multiple m^6^A-modified transcripts. Identification of sepsis-relevant, FTO-dependent target genes and their methylation status will require further validation in future studies using clinical samples.

In conclusion, the present study highlights the role of FTO in regulating macrophage and neutrophil functions, thereby contributing to the understanding of immune mechanisms underlying sepsis. The present results may facilitate further investigation into the immunopathogenesis of sepsis and the potential therapeutic implications of targeting FTO.

## Data Availability

The datasets presented in this study can be found in online repositories. The names of the repository/repositories and accession number(s) can be found in the article/**Supplementary Material**.
