# Supplementary material for: Identification of FTO as a key m6A demethylase linking immune dysregulation to sepsis pathogenesis
Source: Front Immunol. 2026 Feb 18;17:1756059. doi: 10.3389/fimmu.2026.1756059 (PMC12956523; doi:10.3389/fimmu.2026.1756059)
Supplement: Supplementary file 1 [file Image1.pdf]

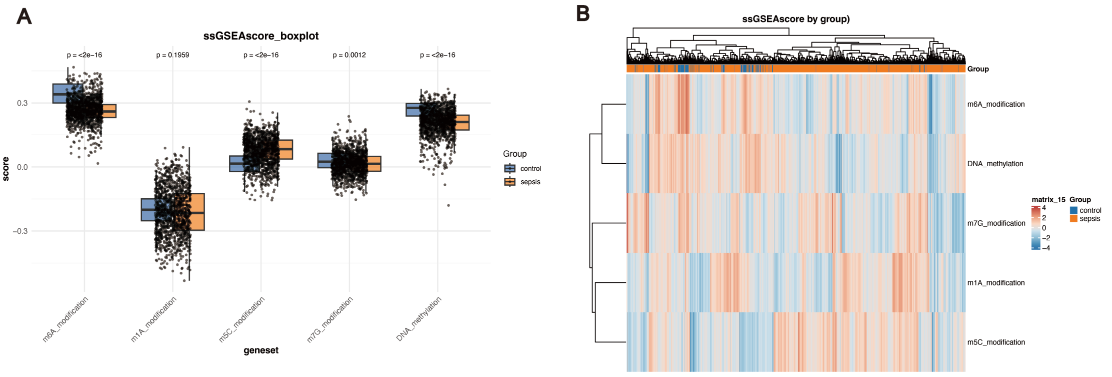

Supplementary Figure 1. Pathway-level comparison of ssGSEA scores between sepsis and control samples. (A) Boxplots showing the distribution of ssGSEA scores for different RNA modification and epigenetic regulatory gene sets, including m6A, m1A, m5C, and m7G RNA modifications, as well as DNA methylation, in sepsis and control groups. (B) Heatmap illustrating the ssGSEA scores of the indicated gene sets across individual samples, with samples grouped by clinical status (sepsis vs control).
