# Supplementary material for: Identification of FTO as a key m6A demethylase linking immune dysregulation to sepsis pathogenesis
Source: Front Immunol. 2026 Feb 18;17:1756059. doi: 10.3389/fimmu.2026.1756059 (PMC12956523; doi:10.3389/fimmu.2026.1756059)
Supplement: Supplementary file 2 [file Image2.pdf]

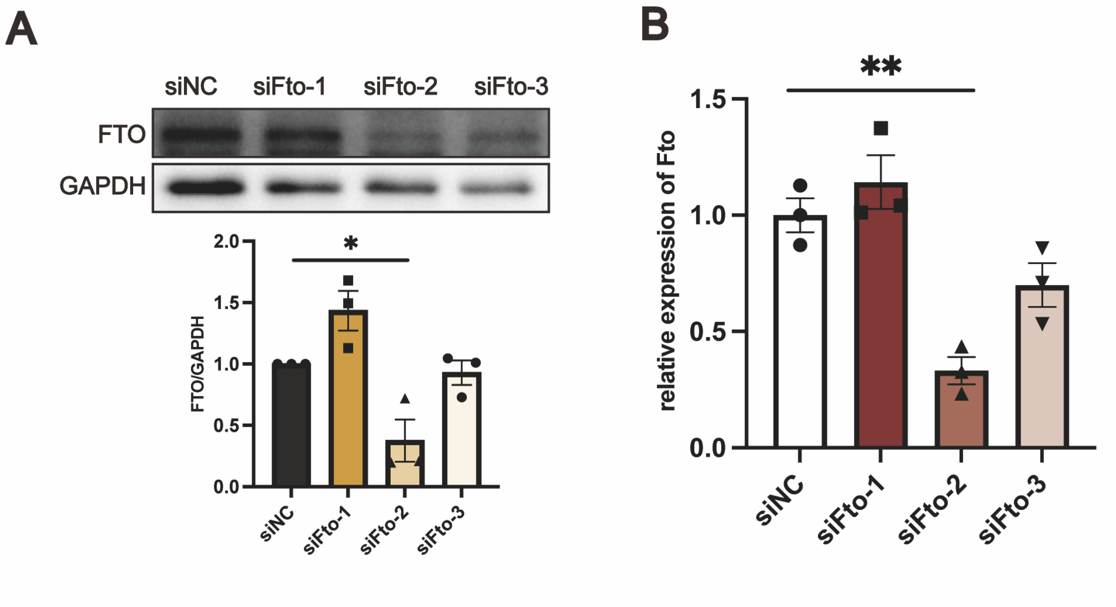

Supplementary Figure 2. (A–B) Validation of FTO knockdown efficiency in RAW264.7 cells transfected with siRNAs.
