# Supplementary material for: Identification of FTO as a key m6A demethylase linking immune dysregulation to sepsis pathogenesis
Source: Front Immunol. 2026 Feb 18;17:1756059. doi: 10.3389/fimmu.2026.1756059 (PMC12956523; doi:10.3389/fimmu.2026.1756059)
Supplement: Supplementary file 3 [file Table1.docx]

**Supplementary Table 1. siRNA sequences.**

| Sequence name | Sequence information | |
| --- | --- | --- |
| mFto-siRNA-1 | Sequence-Sense | GCUGAUGACUGCUGAUUUATT |
|  | Sequence-Antisense | UAAAUCAG CAGUCAUCAGCTT |
| mFto-siRNA-2 | Sequence-Sense | GCACCUACAAGUACUUGAATT |
|  | Sequence-Antisense | UUCAAGUAC UUGUAGGUGCTT |
| mFto-siRNA-3 | Sequence-Sense | GCAUGGACUCCGAUUUCAATT |
|  | Sequence-Antisense | UUGAAAUCG GAGUCCAUGCTT |
| siRNA-NC | Sequence-Sense | UUCUCCGAACGUGUCACGUTT |
|  | Sequence-Antisense | ACGUGACACGUUCGGAGAATT |
