# Supplementary material for: Identification of FTO as a key m6A demethylase linking immune dysregulation to sepsis pathogenesis
Source: Front Immunol. 2026 Feb 18;17:1756059. doi: 10.3389/fimmu.2026.1756059 (PMC12956523; doi:10.3389/fimmu.2026.1756059)
Supplement: Supplementary file 4 [file Table2.docx]

**Supplementary Table 2. Primer sequences.**

| Gene | Forward sequence (5'-3') | Reverse sequence (5'-3') |
| --- | --- | --- |
| *Fto* | TTCATGCTGGATGACCTCAATG | GCCAACTGACAGCGTTCTAAG |
| *Tnfα* | GTGCCTATGTCTCAGCCTCTTCTC | TGGTTTGTGAGTGTGAGGGTCTG |
| *IL1b* | TTCAGGCAGGCAGTATCACTC | GAAGGTCCACGGGAAAGACAC |
| *IL6* | TAGTCCTTCCTACCCCAATTTCC | TTGGTCCTTAGCCACTCCTTC |
| *Gapdh* | TTCACCACCATGGAGAAGGC | GGCATGGACTGTGGTCATGA |
