# Supplementary material for: Identification of FTO as a key m6A demethylase linking immune dysregulation to sepsis pathogenesis
Source: Front Immunol. 2026 Feb 18;17:1756059. doi: 10.3389/fimmu.2026.1756059 (PMC12956523; doi:10.3389/fimmu.2026.1756059)
Supplement: Supplementary file 5 [file Table3.docx]

**Supplementary Table 3. Model performance of the 14 classifiers in training set.**

| Models | Sensitivity | Specificity | Accuracy | PPV | NPV | F1 | Youden's index |
| --- | --- | --- | --- | --- | --- | --- | --- |
| RandomForest | 0.979381443298969 | 0.940298507462687 | 0.969348659003831 | 0.979381443298969 | 0.940298507462686 | 0.979381443298969 | 0.919679950761656 |
| GradientBoosting | 0.984536082474227 | 0.91044776119403 | 0.96551724137931 | 0.969543147208122 | 0.953125 | 0.976982097186701 | 0.894983843668257 |
| SVM_Kernel | 0.984536082474227 | 0.985074626865672 | 0.984674329501916 | 0.994791666666667 | 0.956521739130435 | 0.989637305699482 | 0.969610709339898 |
| LogisticModel | 0.958762886597938 | 0.925373134328358 | 0.950191570881226 | 0.973821989528796 | 0.885714285714286 | 0.966233766233766 | 0.884136020926297 |
| NeighborMethod | 0.984536082474227 | 0.970149253731343 | 0.980842911877395 | 0.989637305699482 | 0.955882352941177 | 0.987080103359173 | 0.95468533620557 |
| PLSModel | 0.979381443298969 | 0.970149253731343 | 0.977011494252874 | 0.989583333333333 | 0.942028985507246 | 0.984455958549223 | 0.949530697030312 |
| BoostingMethod | 0.989690721649485 | 0.925373134328358 | 0.973180076628352 | 0.974619289340101 | 0.96875 | 0.982097186700767 | 0.915063855977843 |
| NeuralNet | 0.984536082474227 | 0.970149253731343 | 0.980842911877395 | 0.989637305699482 | 0.955882352941177 | 0.987080103359173 | 0.95468533620557 |
| BayesMethod | 0.93298969072165 | 0.805970149253731 | 0.900383141762452 | 0.93298969072165 | 0.805970149253731 | 0.93298969072165 | 0.738959839975381 |
| DiscriminantModel | 0.974226804123711 | 0.970149253731343 | 0.973180076628352 | 0.989528795811518 | 0.928571428571428 | 0.981818181818182 | 0.944376057855055 |
| Lasso | 0.984536082474227 | 0.970149253731343 | 0.980842911877395 | 0.989637305699482 | 0.955882352941177 | 0.987080103359173 | 0.95468533620557 |
| AdaptiveBoosting | 0.984536082474227 | 0.955223880597015 | 0.977011494252874 | 0.984536082474227 | 0.955223880597015 | 0.984536082474227 | 0.939759963071242 |
| CATBoost | 0.994845360824742 | 0.970149253731343 | 0.988505747126437 | 0.98974358974359 | 0.984848484848485 | 0.992287917737789 | 0.964994614556085 |
| LightGBM | 1 | 1 | 1 | 1 | 1 | 1 | 1 |
