# Supplementary material for: Identification of FTO as a key m6A demethylase linking immune dysregulation to sepsis pathogenesis
Source: Front Immunol. 2026 Feb 18;17:1756059. doi: 10.3389/fimmu.2026.1756059 (PMC12956523; doi:10.3389/fimmu.2026.1756059)
Supplement: Supplementary file 6 [file Table4.docx]

**Supplementary Table 4. Model performance of the 14 classifiers in validation set 1.**

| Models | Sensitivity | Specificity | Accuracy | PPV | NPV | F1 | Youden's index |
| --- | --- | --- | --- | --- | --- | --- | --- |
| RandomForest | 1 | 0 | 0.75 | 0.75 | NA | 0.857142857142857 | 0 |
| GradientBoosting | 1 | 0 | 0.75 | 0.75 | NA | 0.857142857142857 | 0 |
| SVM_Kernel | 1 | 0 | 0.75 | 0.75 | NA | 0.857142857142857 | 0 |
| LogisticModel | 1 | 0 | 0.75 | 0.75 | NA | 0.857142857142857 | 0 |
| NeighborMethod | 1 | 0 | 0.75 | 0.75 | NA | 0.857142857142857 | 0 |
| PLSModel | 1 | 0 | 0.75 | 0.75 | NA | 0.857142857142857 | 0 |
| BoostingMethod | 1 | 0 | 0.75 | 0.75 | NA | 0.857142857142857 | 0 |
| NeuralNet | 0.98 | 0 | 0.735 | 0.746192893401015 | 0 | 0.847262247838617 | -0.02 |
| BayesMethod | 1 | 0 | 0.75 | 0.75 | NA | 0.857142857142857 | 0 |
| DiscriminantModel | 1 | 0 | 0.75 | 0.75 | NA | 0.857142857142857 | 0 |
| Lasso | 1 | 0 | 0.75 | 0.75 | NA | 0.857142857142857 | 0 |
| AdaptiveBoosting | 1 | 0 | 0.75 | 0.75 | NA | 0.857142857142857 | 0 |
| CATBoost | 1 | 0 | 0.75 | 0.75 | NA | 0.857142857142857 | 0 |
| LightGBM | 1 | 0 | 0.75 | 0.75 | NA | 0.857142857142857 | 0 |
